# Insights into N6-methyladenosine and programmed cell death in cancer

**DOI:** 10.1186/s12943-022-01508-w

**Published:** 2022-01-28

**Authors:** Li Liu, Hui Li, Dingyu Hu, Yanyan Wang, Wenjun Shao, Jing Zhong, Shudong Yang, Jing Liu, Ji Zhang

**Affiliations:** 1 Department of Clinical Laboratory, Shenzhen Traditional Chinese Medicine Hospital, Shenzhen, 518033 Guangdong China; 2 The First Affiliated Hospital, Department of Rheumatology, Hengyang Medical School, University of South China, Hengyang, 421001 Hunan China; 3 Hunan Province Key Laboratory of Basic and Applied Hematology, Molecular Biology Research Center & Center for Medical Genetics, School of Life Sciences, Central South University, Changsha, 410078 Hunan China; 4 Department of Dermatology, Hunan Key Laboratory of Skin Cancer and Psoriasis, Hunan Engineering Research Center of Skin Health and Disease, Xiangya Clinical Research Center for Cancer Immunotherapy, Xiangya Hospital, Central South University, Changsha, 410008 Hunan China; 5 The First Affiliated Hospital, Department of Hematology, Hengyang Medical School, University of South Chinal, Hengyang, 421001 Hunan China

**Keywords:** N6-methyladenosine, Cancer, Apoptosis, Autophagy, Pyroptosis, Ferroptosis, Necroptosis

## Abstract

N6-methyladenosine (m6A) methylation, the most common form of internal RNA modification in eukaryotes, has gained increasing attention and become a hot research topic in recent years. M6A plays multifunctional roles in normal and abnormal biological processes, and its role may vary greatly depending on the position of the m6A motif. Programmed cell death (PCD) includes apoptosis, autophagy, pyroptosis, necroptosis and ferroptosis, most of which involve the breakdown of the plasma membrane. Based on the implications of m6A methylation on PCD, the regulators and functional roles of m6A methylation were comprehensively studied and reported. In this review, we focus on the high-complexity links between m6A and different types of PCD pathways, which are then closely associated with the initiation, progression and resistance of cancer. Herein, clarifying the relationship between m6A and PCD is of great significance to provide novel strategies for cancer treatment, and has a great potential prospect of clinical application.

## Introduction

To date, out of more than 160 chemical modifications, N6-methyladenosine, a methylation modification of the sixth adenine (A) nitrogen, is the most common internal RNA modification occurring at the post-transcriptional level, especially in eukaryotic mRNAs [1–4]. M6A methylation frequently occurs around stop codons and 3′ untranslated regions (3′-UTRs) and is modulated by three regulators: writers, readers and erasers [5], subsequently affecting related physiological functions and pathological processes, particularly in tumorigenesis [6–8].

In most cases, m6A plays oncogenic roles in the tumorigenesis and development of cancers, such as gastric cancer (GC), bladder cancer and hepatocellular carcinoma (HCC) [9–12]. However, it also has been reported that inhibiting METTL14 (methyltransferase-like protein 14)-mediated m6A abundance facilitates the development of bladder tumor-initiating cells (TICs) [13]. Whether m6A methylations exhibit anticancer or protumoral effects remain controversial, which may depend on the various characteristics of cancer, such as the intratumoral microenvironment [14, 15]. As more attention has been given to its role in the tumor process, m6A methylations have been increasingly investigated in ovarian cancer [16], colorectal cancer [17], breast cancer [18], and so on. Moreover, several lines of evidence have revealed the essential roles played by m6A RNA modifications (i.e., m6A epitranscriptome) in drug resistance [19], NAFLD development [20, 21], type 2 diabetes [22] and the occurrence of viral hepatitis [23–25], as well as malignant hematopoiesis [26].

Emerging evidence suggests that the combination of apoptosis with other forms of programmed cell death, such as autophagy, pyroptosis, necroptosis, NETosis and ferroptosis, is significantly required for the maintenance of normal cell cycle and tissue homeostasis, and responding in an actively regulated manner to either internal or external threats [27–32]. Programmed cell death (PCD) played a fundamental role in an organism’s balance mechanism by which stressed, damaged, malignant or infected cells were lysed and effectively cleared [33, 34]. Studies have shown that distinct types of cell death are not independent of each other and share a coordinated system. When one pathway is abnormally defective, other regulatory mechanisms will ensure the process of cell death. Importantly, malfunctioning PCD pathways are related to the occurrence and development of diseases [33–35].

As the most prevalent epigenetic modification on mRNAs and noncoding RNAs, m6A methylation influences RNA metabolism, thus negatively controlling protein expression in a posttranscriptional manner; moreover, PCD elicits cell death via complicated cascades of transcriptional alterations and posttranslational protein modifications [32, 36]. Over the past years, mounting evidence has proven that m6A may regulate gene expression, thus affecting diverse programmed cell death processes.

### Methods for m6A analysis

After the discovery of the restriction-modification (R-M) system in the 1960s, m6A marks were first identified in bacterial DNA and extensively studied in eukaryotic DNA and RNA [37–40], which further promoted the related research on m6A in RNAs. However, due to the lack of available methods to define its position in RNA, the biological function of m6A remained largely elusive. It was not until 2011 that the identification of demethylase, fat mass and obesity-associated protein (FTO) directly promoted the development of m6A analysis methods, which established the view of m6A as a dynamic and highly conserved post-transcriptional modification that contains a consensus motif termed “RRACH” in methylated-m6A RNAs [41–44]. To date, many new m6A-related technologies have been developed. For example, MeRIP-m6A-seq allows a global view of ubiquitous RNA peaks as the first protocol. This technique pulls down m6A-containing RNA fragments through specific anti-m6A antibody. Most of the m6A sites in the 3′- UTR from the human transcriptome have been identified. However, the size of the RNA fragment pulled down leads to limited resolution [41, 45], photo-cross-linking-assisted m6A sequencing (PA-m6A-seq) combines antibody-based immunoprecipitation to improve the resolution of m6A profiling, which realizes further breakthroughs in locating m6A sites [46], and matrix-assisted laser desorption/ionization time of flight mass spectrometry (MALDI-TOF-MS) purposefully detects m6A mutations in the corresponding miRNAs. This method alkylates the N1 of adenines in RNA with dimethylsulfate followed by sequential MS analysis [47]. Due to the quickly evolving m6A detection method, the panorama of m6A in cancer is expected to apply to cancer diagnosis and molecular classifications in the future.

### M6A regulators

**Fig. 1:**
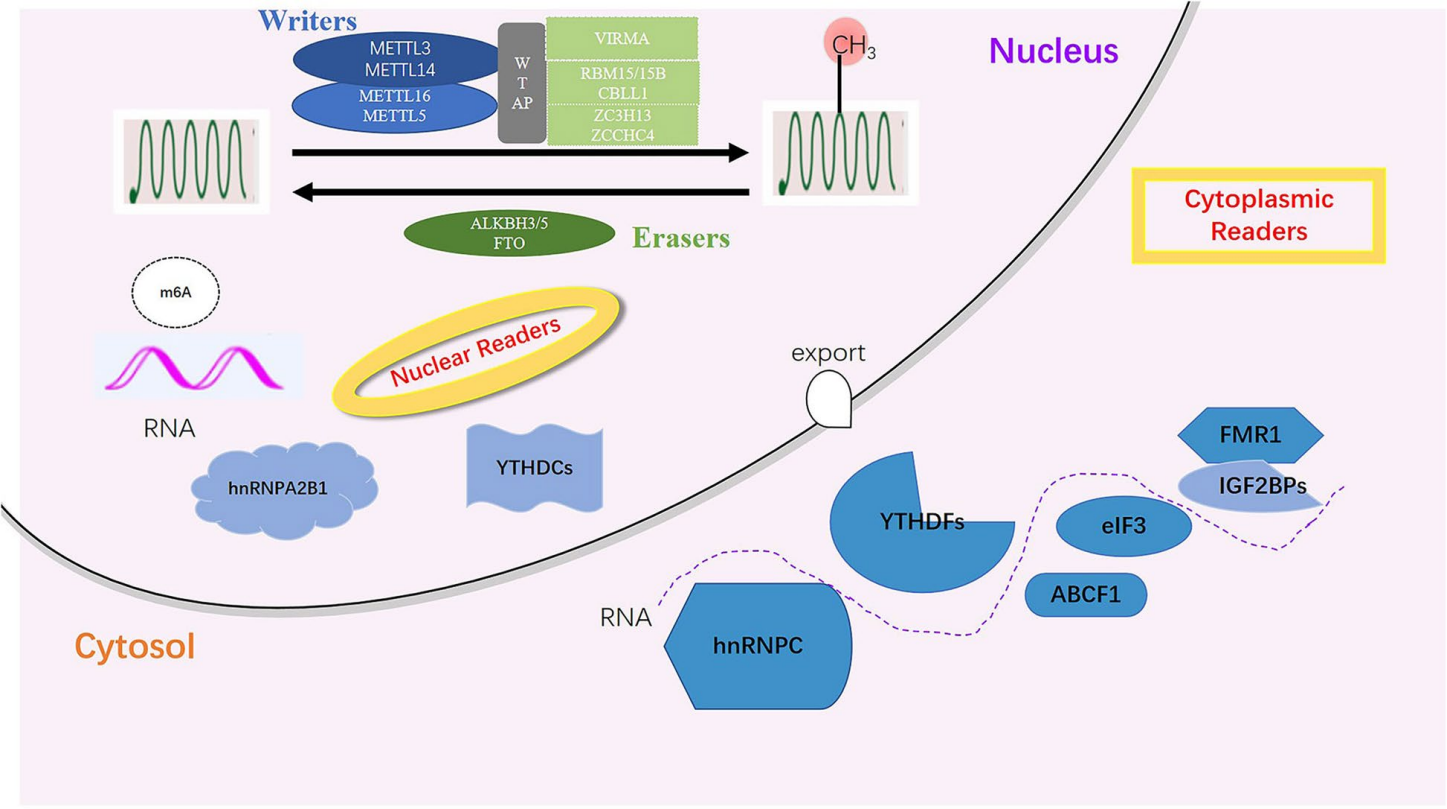
M6A regulators. M6A is deposited by writers, removed by erasers, and recognized by readers

M6A writers, readers and erasers are involved in the initiation, recognition and removal of coding and noncoding RNAs (ncRNAs), respectively (Fig. 1). The detailed regulation of m6A is as follows: m6A writers (METTL3, METTL14, METTL16, WTAP, RBM15/15B, VIRMA, ZC3H13) install m6A sites in RNAs, paramountly, forming a methyltransferase complex (MTC) between methyltransferase-like protein 3 (METTL3) and METTL14. The former mainly catalyzes the transfer of methyl groups in S-adenosyl methionine (SAM) to adenine bases in RNA, whereas the latter stabilizes the conformation of MTC and appraises precise RNA sequences, with additional subunits contributing to the activity and specificity of this complex [48–51]. Wilms tumor 1-associating protein (WTAP) recruits METTL3 and METTL14 into nuclear speckles, promoting RNA-binding capability of m6A methyltransferase. WTAP and METTL3 regulate various genes related to transcription and RNA processing [52]; RNA binding motif protein 15/15B(RBM15/15B) directs METTL3-METTL14 heterodimer to specific RNA sites [53, 54]; Vir like m6A methyltransferase associated (VIRMA) is reported to recruit the MTC and interact with polyadenylation cleavage factors CPSF5 and CPSF6 [55]; Zinc finger CCCH-type containing 13(ZC3H13) keeps MTC location in a WTAP-binding fashion [56]; In comparison with MTC, METTL16 preferentially mediates m6A epitranscriptomic modification of U6-snRNA in a divergent sequence and structure setting [57].

In response to external forces, m6A is identified by diverse readers (YTH domain family, IGF2BPs, HNRNP family, eIF3), triggering a series of downstream biological responses. YTH domain containing 1(YTHDC1) promotes RNA processing and export [58–60]; YTHDC2 enhances target mRNA translation incidence through reducing its abundance [61, 62]; YTH domain family protein 1 (YTHDF1) acts as a translation promoter while YTHDF2 degradation accelerates the downregulation of mRNA levels, and YTHDF3 coordinates with these proteins synergistically, playing an assistant role in target mRNA [63–66]. Insulin-like growth factor 2 mRNA-binding proteins (IGF2BP1/2/3) stabilizes mRNA and promotes translation with eukaryotic translation initiation factor 3 (eIF3) [67, 68]. Heterogeneous nuclear ribonucleoprotein A2/B1 (HNRNPA2/B1) facilitates pri-miRNA processing [69]. In addition, HNRNPC/G interacts with m6A-modified mRNA and affects its enrichment and splicing, generating a phenomenon termed the “m6A switch” [70, 71]. Finally, m6A erasers, FTO and ALKBH3/5, reverse m6A and promote RNA demethylation [72, 73]. These results have revealed how these enzymes and binding proteins modulate m6A-methylated RNAs. It is noted that these “effectors” are not independent components. Furthermore, homeostasis of m6A chemical sites on RNAs is retained in a context-reliant manner, thus impacting gene expression [74].

### M6A modifications in RNA metabolism

As mentioned above, m6A methylations were involved in mRNA metabolism by an expanding list of m6A readers and m6A writer-complex components, as well as erasers, exerting effects on mRNA splicing, translation, and decay [75]. Notably, as described by Berulava et al., transcripts mainly carry m6A modifications around the translation end sites, 5′-UTR or 3′-UTR and coding sequences (CDS). Depending on its location, m6A methylation sites might determine the functional consequences, leading to intricate influences on the mRNA. Specifically, m6A-containing transcripts in 5′-UTR and/or CDS are associated with energy metabolism, mitochondrial function and intracellular pathways, while the majority of transcripts methylated in the 3′-UTR are linked to more specific metabolic processes, including “acetyl-CoA or glycerol biosynthesis” and “positive regulation of protein dephosphorylation”. However, the detailed mechanism remains elusive [76].

**Fig. 2:**
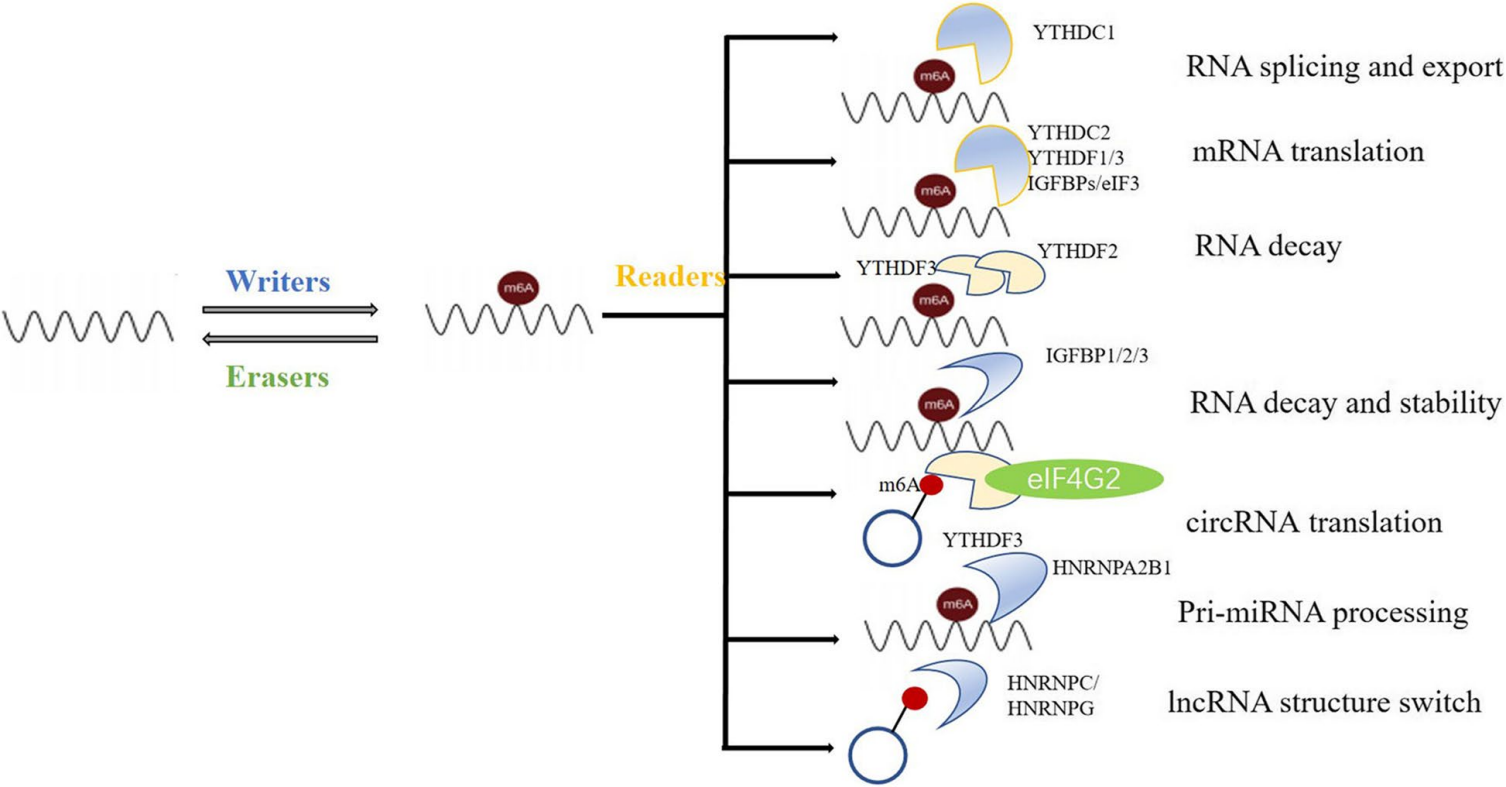
M6A in RNA metabolism. M6A is almost involved in the whole process on mRNA, containing splicing, translation and degradation, what’s more, m6A plays important role in ncRNAs metabolism through sponging various readers, directly or indirectly. The latter requires certain adaptors, such as eIF4G2, DCGR8

Accumulating evidences have shown that m6A decorations also account for indispensable parts in ncRNA processing [77]. The effects of m6A on miRNAs are mediated by a series of m6A regulatory proteins. For example, steady-state levels of several miRNAs, such as let-7e, miR-25, miR-93, miR-126, miR-221/222, and miR-4485, could be affected by altered METTL3, thus facilitating miRNA biogenesis [78]. Similar to METTL3, METTL14, another m6A writer, can also govern the expression of miRNAs with m6A peaks [79]. In addition to the two core compositions of MTC, m6A readers participate in miRNA biogenesis and maturation, especially HNRNPA2B1 [69, 80–82]. The FTO and NOP2/Sun domain family (NSun2, a methyltransferase) play an inhibitory role in m6A-mediated miRNA regulation [83, 84]. Additionally, previous studies indicated the role of m6A readers on circRNAs [85–88]. In addition, m6A is actively involved in the interactions between lncRNAs and other RNAs, such as miRNAs [89], controlling many aspects of gene expression and cellular biology. M6A-snRNAs catalyze pre-mRNA splicing and biogenesis, while m6A-rRNAs manipulate target transcript translation [90, 91], representing typical cases of ncRNA-mRNA interactions. Accordingly, m6A is involved not only in mRNA biogenesis and functions but also in miRNA maturation, circRNA translation, and RNA-RNA interplay. Collectively, the multiple biofunctions of RNAs that are highly m6A-modified, many researchers have reached a consensus on whether and how ncRNAs or mRNA are greatly affected by m6A regulators (Fig. 2). However, the effects should have been virtually made to investigate the specific proof-of-principle of m6A depositions on mRNA and the role of m6A regulators in the transcriptome of ncRNAs.

### M6A and programmed cell death

**Fig. 3:**
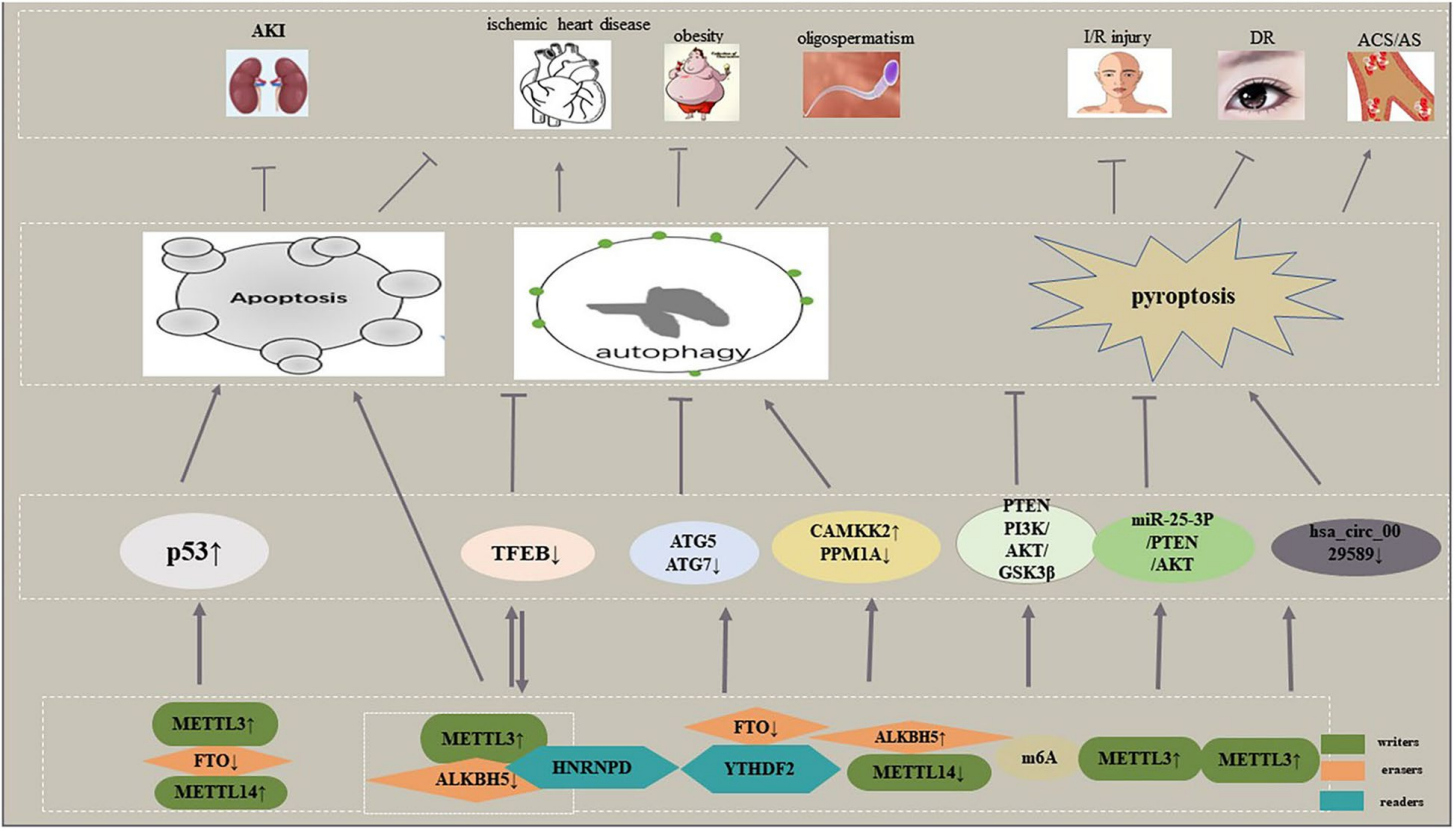
M6A and programmed cell death in non-cancer diseases. Enhanced m6A promotes apoptosis to inhibit the development of AKI and ischemic heart diseases. The latter can be aggravated by inhibition of TFEB-mediated autophagy, and TFEB can regulate METTL3 and ALKBH5 expression. M6A regulators suppress the progression of obesity and oligospermatisms via autophagy. M6A regulates PTEN/PI3K/AKT/GSK3βto inhibit pyroptosis, thus fighting against I/R injury. METTL3 upregulation modulates the development of DR and AS/ACS through pyroptosis

Since it was first identified in apoptosis through modulating gene expression, m6A posed by an increasing number of studies is interplayed with almost all types of PCD pathways. Here, we mainly discuss the relevance between m6A and cell apoptosis, autophagy, pyroptosis, and ferroptosis, as well as necroptosis (Fig. 3). There is no doubt that association of m6A and PCD pathways will provide new insights into the management of related diseases. It is worth mentioning that the interconnectivity among various PCD types significantly dictates cell fate. For instance, autophagy can induce necroptosis.

#### M6A and apoptosis

Numerous studies have found biomarker value in the relevance of m6A and apoptosis. As summarized by Chen et al., m6A induces apoptosis via four mechanisms: governing apoptosis-related genes, silencing methylating or demethylating enzyme genes, and reducing transcripts mediated by Ythdf2 [92]. More reader proteins have been considered activators resulting in apoptosis based on recent reports. According to machine learning and GSEA analysis, the YTHDC2 gene, ranked the highest among 10 genes in experimental models, may increase the translation efficiency of Smc3 (target *YTHDC2* in spermatogenesis) and stimulate the significant elevation of apoptosis-associated genes sush as caspase 3, Bcl-2, and Bid in neck squamous cancer cells, implying better overall survival (OS) in contrast to other regulatory genes [93]. However, the underlying mechanism has not yet been elucidated. In a recent study investigating lncRNAs harboring m6A, LNC942 directly recruited METTL14, a core member of m6A methyltransferase complexes, accompanied by elevated epigenetic m6A methylation modification levels in breast cancer cells, subsequently accelerating cell proliferation and colony formation and decreasing cellular apoptosis rates through LNC942-METTL14-CXCR4/CYP1B1 signaling axis [94]. Epigenetically, FTO depletion via shRNA lentiviral infection manipulates the downstream proapoptosis gene BNIP3 via a YTHDF2-independent mechanism, resulting in breast cell apoptosis in vivo and in vitro [95]. Similar results observed by Zhou et al. indicated that silencing FTO enhances p53 mRNA and protein levels, aggravating excessive apoptosis after cisplatin-induced acute kidney injury (AKI) [96]. As shown by Song et al. they demonstrated that when cardiomyocytes were subjected to hypoxia/reoxygenation, induced METTL3, but not METTL14, accounting for abnormal m6A formation, finally enhanced cell apoptosis; ALKBH5, rather than FTO, executing its demethylation tasks, ultimately repressed apoptosis [97]. Functioning as an apoptotic activator, m6A seems to be regarded as an inhibitor as well. For instance, METTL3 knockdown at the post-transcriptional level recovered the expression of ZNF750 by maintaining mRNA stability, significantly increasing the proportion of apoptotic cells dependent on FGF14 in NPC cells, and vice versa [98]. These results revealed that m6A is actively involved in cell apoptosis and probable to evaluate disease outcomes. Nevertheless, it is still possible that m6A-mediated apoptosis could have additional effects that remain to be discovered.

#### M6A and autophagy

Autophagy, an emerging PCD pathway, is tightly influenced by distinct stimulators and suppressors through frequently altered autophagy-related (ATG) proteins and transcription factors. In 2018, the relationship between autophagy and m6A was first described. ULK1 is a protein kinase that is motivated by autophagy. The m6A distribution within its transcripts can be abrogated by FTO, thus promoting the production of ULK1 protein, LC3BII and autophagic flux owing to a YTHDF2-regulated mechanism, which symbolizes the initiation of autophagy [99]. M6A serves as an inhibitor that enhanced METTL3 reduces autophagic flux via regulating TFEB levels and transcriptional activity, while ALKBH5 does the opposite effect in H/R-treated cardiomyocytes. Given both the contradictory role in TFEB and that it reacts on these m6A enzymes toward completely distinct directions, ALKBH5 expression is upregulated, and METTL3 is not [97]. Autophagy has been known to regulate adipose mass and adipogenesis; FTO was firstly familiar for regulating lipid metabolism. It is striking to confirm that FTO reduction, with YTHDF2-mediated mRNA decay and ATG5 and ATG7-CEBPB signaling transmitted, obviously decreases the quantity of steady-state autophagosomes—a positive correlation between FTO and autophagy, compared to the control cells. Furthermore, attenuated fat accumulation via anti-autophagy activated by FTO deficiency is of critical functional significance for the prevention and elimination of the increasingly detrimental effects of obesity [100]. In contrast, FTO upregulation caused by low-level arsenic exposure impaired p62-mediated autophagy and autophagic degradation and formed a positive feedback loop to accumulate FTO, thus facilitating tumorigenesis in keratinocytes. This article found that IGF2BPs could reverse the effect of FTO and need further investigation [101].

With m6A engaged in the differentiation of embryonic stem cells (ESCs), sex determination, and spermatogenesis, what about those m6A modulators who are implicated in the differentiation of Leydig cells (LCs) and testosterone synthesis? The answer is that METTL14 and ALKBH5, one reduction and the other increment lead to reduced m6A levels, resulting in enhanced CAMKK2 (calcium/calmodulin-dependent protein kinase 2, beta) transcript but decreased PPM1A (protein phosphatase 1A, magnesium dependent, alpha isoform) protein, and intensifying the autophagy phenotype, e.g., upregulated LC3 puncta, based on (AMP-activated protein kinase) AMPK-ULK1 signals, giving assistance to the production of testosterone, irrespective of LCs age. These findings will facilitate an understanding of the importance of m6A in autophagy for the purpose of targeting azoospermatism and oligospermatism patients [102].

In parallel, autophagy also plays a role in m6A modifications. Recently, compelling evidence has proven that inhibition of FTO, targeting PD-1, CXCR4, and SOX10 in an m6A/YTHDF2-mediated manner, threatens to interfere with the malignant transformation of melanoma cells and growth of melanoma, and downregulation of the essential autophagy genes ATG5 or ATG7 reduces starvation-induced FTO and PD-1 expression, as well as NF-κB activity under metabolic stress, suggesting that upregulated FTO advancing melanoma tumorigenesis is induced by autophagy and the NF-κB pathways [103]. These findings highlight the interplay between autophagy and m6A modulators, pointing to diversified functions according to the state of the body. Despite the increasing appreciation of the biological significance regarding m6A modifications, the global effects of m6A regulators on the transcription and translation of more autophagy-related genes, combined with molecular determinants conferring RNA specificity, remain poorly understood. In turn, it may be very interesting to examine whether autophagy can regulate additional m6A components.

#### M6A and pyroptosis

Pyroptosis, a lytic form of cell death whose definitive hallmark genes are NLR pyrin domain containing 3 (NLRP3), apoptotic speck-like protein containing CARD (ASC), cleaved Caspase-1, Gasdermin-D (GsdmD) p30, IL-1β and IL-18, plays a prominent role in response to infection, ultimately fueling inflammation. Phosphatase and tensin homolog (PTEN) is regarded as a classic tumor suppressor. The latest studies have elucidated the regulation of PTEN on pyroptosis in human diseases. Notably, PTEN mRNA, which contains m6A sites, is involved in hypothermia-activated PI3K/Akt/GSK-3β signaling, downregulating the expression of pyroptosis-related proteins, i.e., NLRP3 and ASC, and the secretion of proinflammatory cytokines, further protecting hippocampal neurons against H/R-induced pyroptosis upon hypoxia/reoxygenation (H/R) [104]. The participation of METTL3-mediated m6A in the pyroptosis of diabetic retinopathy (DR) was recently reported. Inhibition of METTL3, in line with miR-25-3p ablation, induced serious high glucose-fueled RPE cell apoptosis and pyroptosis and depressed cell viability by regulating the miR-25-3p/PTEN/Akt signaling cascade in a DGCR8-dependent manner [105]. Beyond doubt, this discovery will bring new opportunities for the future treatment of DR through pointing at METTL3. According to Guo et al., IFN regulatory factor (IRF)-1, an IFN-inducible transcription factor, was validated to promote pyroptosis and inflammation in cultured macrophages from patients with atherosclerosis (AS) and acute coronary syndrome (ACS) by significantly decreasing the expression of hsa_circ_0029589 but elevating its m6A level, along with METTL3 expression. That is to say, m6A modifications perform pivotal parts in IRF-1-induced macrophage pyroptosis [106]. Although investigations on IRF-1 in circRNA from macrophages are still in infancy, this newly discovered regulatory network is expected to provide promising therapeutic options for ACS and AS. As a consequence, these implications critically build a bridge between m6A and pyroptosis-related components, suggesting that m6A modifications on mRNAs and ncRNAs exhibit important functions in cell pyroptosis by influencing the enrichment of NLRP3, ASC, etc. Nevertheless, more research is warranted to explore other RNAs with m6A modifications in pyroptosis.

#### M6A and ferroptosis

Ferroptosis, a novel proinflammatory programmed cell death pathway, which plays a pivotal role in clearing malignant cells, is induced by the suppression of the xCT/GSH/GPX4 axis and mainly characterized by iron accretion and lipid peroxidation, accompanied by compression of mitochondrial membrane densities, reduction or disappearance of mitochondria crista and rupture of the outer mitochondrial membrane [107]. Recent studies have unraveled the relationship between m6A and ferroptotic cell death. Exosomes were released by tumor cells, and miR-4443 expression was significantly enhanced in this environment. This upregulation is associated with the chemoresistance of NSCLC to cisplatin. Further study discovered that miR-4443 mediated the production of intracellular superoxide, ROS and ferrous iron and the expression of FSP1, inhibiting ferroptosis, which was executed by decreased m6A-modified levels of FSP1 through METTL3 [108]. This finding offered a promising opportunity for increasing the efficacy of cisplatin by diminishing exosomal miR-4443 expression, enhancing FSP1 m6A deposition, and promoting ferroptotic cell death. Additionally, ferroptosis can be induced by suppression of system X_c_^−^, a cystine/glutamate antiporter. Two major subunits of this system are SLC7A11 (solute carrier 7A11) and SLC3A2 (solute carrier 3A2). Ma et al. found that both of these subunits were involved in YTHDC2-regulated ferroptosis in lung adenocarcinoma (LUAD) cells. Mechanistically, YTHDC2 decreased homeobox A13 (HOXA13) mRNA stability by recognizing the m6A site in the 3′-UTR to suppress SLC3A2 expression, thereby inhibiting LUAD antioxidation and further limiting tumor progression. However, the detailed degradation mechanism is unclear. In summary, YTHDC2 can act as an endogenous ferroptosis motivator for LUAD patients to guide clinical therapy [109]. These researches link m6A to ferroptosis; therefore, targeting m6A to induce ferroptosis might be a promising strategy for ferroptosis-based therapy.

#### M6A and necroptosis

Necroptosis is a proinflammation regulated necrosis that generally results from overwhelming intra- and extracellular insults, such as TNFα, the Fas ligand and ROS production. The morphological features are similar to nonregulated necrosis, manifesting as cytoplasmic vacuolization, organelle swelling and membrane rupture, which release DAMPs (damage-associated molecular patterns) to function. Most importantly, necroptosis can establish a cancer immunogenic microenvironment [110]. Lan et al. recently revealed that M2-polarized tumor-associated macrophages (TAMs) and METTL3 in the oxaliplatin-tolerant CRC patient tumor microenvironment (TME) are higher than those in their sensitive counterparts. Further studies revealed that infiltrated M2-TAMs increased the m6A level of TRAF5 mediated by METTL3 and then impaired the necroptosis process, finally causing acquired oxaliplatin tolerance. This study suggests that METTL3 depletion contributes to TRAF5-mediated necroptotic cell death to improve oxaliplatin resistance in CRC patients [111]. Evading apoptosis is an important means of neoplasm progression, and studies have shown that suppression of caspase8 can trigger necroptosis and that m6A regulates necroptosis, thus inducing necroptosis based on m6A regulation opens up a new avenue for anticancer strategies by blocking caspase-dependent apoptosis [112]. As mentioned earlier, the m6A modifier acts as a double-edged sword in modulating various cellular processes related to cell death, which likely hinges on abundant RNA-binding proteins (RBPs), containing m6A reader proteins and nonreader proteins, and their recognition locations. Therefore, it may be promising to identify crosstalk between m6A effectors and nonreader RBPs, or protein recruitment directly to RNAs and involvement in PCD pathways.

### M6A-regulated PCD, tumorigenesis and tumor progression

**Fig. 4:**
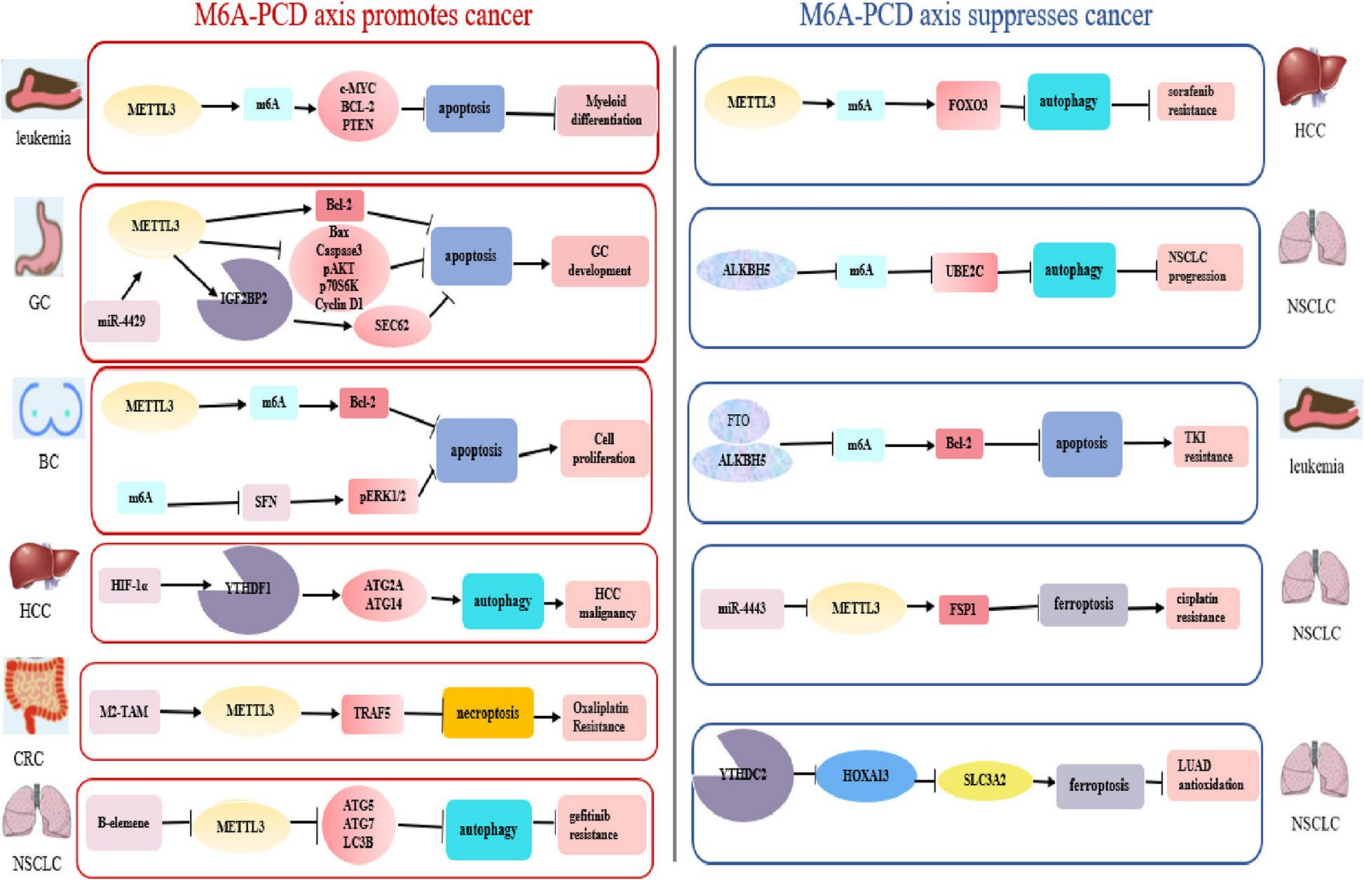
M6A and programmed cell death in cancers. M6A acts as a double-edged sword in the process of the initiation and progression of various cancers via influencing apoptosis, autophagy, ferroptosis and necroptosis processes

As mentioned above, m6A is intensively relative to the initiation and development of various cancers through PCD pathways. M6a-PCD axis acts as a double-edged sword in the pathogenesis and development of various cancers (Fig. 4).

#### M6A-PCD Axis promoting tumorigenesis and tumor progression

At the molecular level, YTHDF1 stimulated by hypoxia-induced HIF-1α was correlated with poor survival in multiple HCC models. MeRIP-seq and proteomics analysis indicated that YTHDF1, an m6A reader, recruited initiation factors and facilitated ribosome loading to promote ATG2A and ATG14 translation through recognition of the m6A site at their CDS, thus mediating autophagy under hypoxia and HCC malignancy [113]. Positive alteration of Bcl-2 is driven by METTL3-installed m6A in breast cancer [114]. Consistently, METTL3 overexpression, together with m6A, increased the enrichment of c-MYC, Bcl-2 and PTEN proteins and undifferentiated cells, reducing apoptotic human myeloid leukemia MOLM13 cells. Pointing to myeloid tumors, these results suggest a potential therapeutic index—inhibiting METTL3 and thus inducing apoptosis [115]. Among the great number of findings, METTL3 was demonstrated to play multiple roles in gastrointestinal cancer development, such as proliferation, invasion, migration, and apoptosis [116]. More importantly, with regard to m6A and apoptosis as revealed by Wang et al. m6A depresses the expression of Bax and active caspase-3 but promotes the synthesis of Bcl-2, thereby countering cancer cell apoptotic pathways. Compared with normal cells, METTL3 increased in gastric cancer cells [117]. The latest findings have confirmed that METTL3 hampered colorectal cancer cell necroptosis to promote oxaliplatin resistance [111]. Furthermore, m6A has an impact on autophagy through regulating autophagy-associated proteins, altering the sensitivity of cancer cells to anticancer drugs [118]. Conspicuously, METTL3 will be realized as a hallmark for cancer initiation and propagation and deserve appreciation.

Sulforaphane (SFN), a dietary phytochemical, could induce apoptosis, nitro-oxidative stress, and genotoxicity and decrease the AKT pathway in breast cancer cells. The stimulation of energy stress by SFN is determined by autophagy activation at both cytostatic and cytotoxic concentrations. Notably, SFN-mediated cell cycle arrest and cellular senescence in cancer cells might be related to suppressed m6A RNA methylation through enhancing the instability of target genes to some extent. Regarding the interaction between SFN-regulated autophagy and apoptosis, previous studies have presented comparatively distinct results: diminished autophagy facilitated apoptotic cell death in prostate, colon and breast cancer cells but functioned independently in SFN-treated pancreatic carcinoma cells [119]. Therefore, exploration of the basal association of autophagy with apoptosis regulated by m6A will assist in the identification of pathogenic mechanisms for carcinogenesis and development. Moreover, m6A is regarded as a promising candidate for cancer therapy since the initiation of malignancies is characterized by an aberrant epigenetic landscape.

#### M6A-PCD Axis inhibiting tumorigenesis and tumor progression

The involvement of extraordinary autophagy-related transcription factors (TFs) in m6A-governed PCD pathways has been reported. Exposed to hypoxia, FOXO3 is such one that downregulated METTL3 raised resistance to sorafenib in HCC by decreasing its mRNA stabilization in a YTHDF1-reliant fashion in that the concomitant m6A decrease enhanced the level of autophagosomes and LC3 accumulation [120]. In leukemia, some studies displayed a negative correlation between m6A and apoptosis—Bcl-2 overexpression evoked by FTO and ALKBH5 upregulation fights against apoptosis, resulting in resistance of leukemia cells to tyrosine kinase inhibitors (TKIs) [114]. METTL3 and YTHDC2 are both ferroptosis inducers that the former could raise cisplatin efficacy in NSCLC and the latter could inhibit lung adenocarcinoma tumorigenesis [108, 109].

Intriguingly, UBE2C, mediated by ALKBH5, links apoptosis (type I PCD) with autophagy (type II PCD), which coregulates autophagic apoptosis in cancer cells. Increased UBE2C expression elevated autophagic phenotypes and drastically governed cancer cell invasive growth, EMT panels and apoptotic cell death in NSCLC, revealing the roles of motivation of autophagy genes in autophagic and apoptotic functions [121].

In accordance with these findings, the interplay between m6A and PCD in the process of tumorigenesis and progression requires attentions, and its investigation will not only contribute to understanding the cancer epigenome, including the m6A signaling network, but also to identifying anticancer targets. More researches on m6A and other PCD signaling pathways in various cancers are urgently needed in the future.

### Clinical application of the target-based m6A-PCD Axis

Emerging data have indicated that m6A modifiers are generally dysregulated in various diseases, characterized by aberrant global m6A abundance, gene expression levels of m6A writers, erasers and readers, and mutations in m6A sites, as well as epitranscriptome changes upon external cues, thereby providing promising biomarkers for clinical diagnosis, treatment and prognosis [122]. Given the multiple functions of m6A-regulated proteins in PCD, making elaboration on how they interact in pathological processes will be propitious to discover specific potential biomarkers and therapeutic targets for cancer diagnosis, treatment and prognostic evaluation, as well as drug resistance (Table 1). In particular, by focusing on the links between m6A and cell apoptosis, autophagy, ferroptosis and necroptosis in cancer, we cannot ignore the potential value of m6A in antitumor actions. In summary, the m6A-mediated PCD axis has become a vital mechanism during pathological processes. As RNAs contain multiple m6A sites in PCD pathways, targeting this interplay shows more diagnostic and therapeutic advantage and potential than targeting a single m6A. Concurrently, by expanding our view of m6A and m6A-regulated PCD, we will broaden our understanding of a variety of m6A protein molecular functions and their application as prognostic indicators of clinical severity.

**Table 1:** The clinical application of m6A and PCD

M6A associated molecules	Types of programmed cell death pathways	Molecular mechanisms	The corresponding cancers	Clinical application	Ref.
FTO	Apoptosis	Increased MZF1 expression	LUSC	Prognosis and therapy	[123]
METTL3 and IGFBP2	Apoptosis	Promoted the stability of SEC62 mRNA	Gastric cancer	Prevention and management	[124]
METTL3 and YTHDF2	Apoptosis	Mediated LHPP and NKX3–1 mRNA	Prostate cancer	Diagnosis and therapy	[125]
HNRNPC	Apoptosis	Downregulated miR-21 and AKT phosphorylation	Ovarian cancer	Prognosis	[126]
METTL3	Autophagy	Stimulated autophagy of ARHGAP5-AS1 or destabilizing ARHGAP5	Gastric cancer	Anti- chemoresistance and prognosis	[127]
METTL3	Apoptosis	Impinged on critical cellular processes	Cervical cancer	Radio/ chemo-resistance	[128, 129]
METTL14	Apoptosis	Targeted CXCR4 and CYP1B1	Breast cancer	Diagnosis, therapy and prognosis	[94]
WTAP	Apoptosis	Affected PI3K/AKT signaling	AML	Prognosis	[130]
WTAP	Apoptosis	Regulated DUSP6	NKTCL	Drug resistance	[131]
YTHDF2	Apoptosis	Reduced the half-life of Tnfrsf2	AML	Therapy	[132]
ALKBH5	Apoptosis	Reduced m6A-methylated pre-miR-181b-1 and YAP	Osteosarcoma	Therapy	[133]
ALKBH5 And YTHDF2	Apoptosis		Pancreatic cancer	Diagnosis and therapy	[134]
FTO	Autophagy	Augmented m6A demethylation of ATG5 and ATG7	Ovarian cancer	Diagnosis and therapy	[135]
FTO	Apoptosis	Caused inhibition of MYC/CEBPA signaling	Glioma	Anti-cancer activity	[136]
FTO	Apoptosis	Reduced hypomethylation and Bcl-2 transcripts	AML	Drug resistance	[137]
M6A	Apoptosis	Regulated the Daple/β-catenin/ABCC9 signaling pathway	NPC	Drug resistance	[138]
RBM15	Apoptosis	Upregulated TMBIM6 m6A modification	LSCC	Therapy and Prognosis	[139]
FTO	Autophagy	Reduced m6A methylation of the critical protumorigenic melanoma cell-intrinsic genes containing PD-1(PDCD-1), CXCR4 and SOX10 and maintaining their mRNA stability	Melanoma	Immune therapy resistance	[103]
M6A writers	Apoptosis	Correlated with drugs which targeted oncogenic related pathways, such as MAPK, EGFR, and mTOR signaling pathways	Colorectal cancer	Immune therapy resistance	[140]

*LUSC* lung squamous cell carcinoma, *NKTCL* Natural killer/T-cell lymphoma, *DUSP6* dual-specificity phosphatases 6, *YAP* Yes-associated protein 1

#### Diagnostic potential

As performed by RNA-seq assay in pancreatic cancer (PC), ALKBH5 overexpression and thus PER1 demethylation altered related apoptotic transcriptome in a YTHDF2-dependent way, which suppressed tumoral growth. Therefore, ALKBH5 and PER1 may be biomarkers for PC diagnosis and therapy [134]. LNC942 acted as a tumor-driver gene in breast cancer cells. Specifically, elevated METTL14 recruited by LNC942 promoted cell proliferation and inhibited cell apoptosis through its target genes like CXCR4 and CYP1B1. It is noteworthy that the METTL14-apoptosis axis plays role in early diagnosis, treatment and prognosis evaluation for breast cancer [94]. As previous work reported, knock-down of YTHDF2 and METTL3 significantly triggered cell apoptosis by mediating LHPP and NKX3–1 mRNA levels, and this finding may contribute to the expansion of potential diagnosis or therapeutic markers of prostate cancer [125]. Additionally, circRAB11FIP1 overexpression augmented m6A demethylation of ATG5 and ATG7 to promote autophagy through sponging FTO to aggravate malignant behavior of ovarian cancer [135].

#### Therapeutic potential

METTL3 is frequently associated with many types of cancers, such as gastric cancer [12], bladder cancer [9], and colorectal cancer [141]. Accordingly, He et al. found that upregulated METTL3 promoted the stability of SEC62 mRNA via IGF2BP1 in GC cell lines. Moreover, miR-4429 might inhibit the expression of SEC62 by controlling METTL3. Furthermore, miR-4429 or silencing SEC62 blocked tumor cell proliferation and facilitated apoptosis, suggesting that miR-4429 and its downstream gene METTL3 are potential targets for gastric cancer prevention and management [124]. In addition, mutant m6A is related to leukemogenesis by governing PCD pathways. In Paris et al.’s AML study, elevated YTHDF2 was found to play a pivotal role in leukemia initiation and propagation. At the molecular level, YTHDF2 helps the functional integrity of leukemic stem cells (LSCs) by reducing the half-life of corresponding m6A targets containing the tumor necrosis factor receptor Tnfrsf2, whose elevation stimulates cell apoptosis in Ythdf2-deficient LSCs. In particular, YTHDF2 deletion contributes to HSC activity, while YTHDF2 does not derail normal hematopoiesis. In accordance, YTHDF2 loss will be a critical target for treating AML through bringing about HSC expansion and promoting myeloid reconstitution, specifically, selectively eradicating malignant LSCs [132]. The demethylase ALKBH5 was downregulated in osteosarcoma, and m6A methylation of pre-miR-181b-1 and YAP-mRNA was upregulated. This promoted osteosarcoma cell growth, migration, and invasion and suppressed cell apoptosis. In contrast, ALKBH5 overexpression had antitumor effects. Therefore, ALKBH5-based m6A demethylation opens up a new strategy for replacement therapy against osteosarcoma through m6A-correlated posttranscriptional regulation of pre-miRNA-181b-1 and Yes-associated protein 1 (YAP) [133]. R-2-Hydroxyglutarate (R-2HG), usually an oncogenic metabolite, exhibits anticancer activity in leukemia and glioma patients with IDH1/2 mutations. In terms of mechanism, R-2HG elevated global m6A by inhibiting FTO in R-2HG-sensitive cells, causing inhibition of MYC/CEBPA signaling and thus resulting in cancer cell cycle arrest and apoptosis. S-2HG showed the same effect. The inhibition of FTO enzymatic activity might enhance the response to chemotherapeutic agents. Overall, this work provided previously unrecognized insights into 2HG-type molecules or selective FTO suppressors, alone and specifically combined with other agents, for example, hypomethylating AZA, which holds therapeutic potential in IDH wild-type tumors with high FTO [136].

#### Prognostic potential

As elucidated by Liu et al. abnormal m6A motifs in lung squamous cell carcinoma (LUSC) were attributed to the FTO gene, which reduced its m6A levels and mRNA stability, contributing to increased MZF1 expression and thus oncogenic impacts. Furthermore, FTO deletion significantly promoted the apoptosis and repressed the proliferation and invasion of L78 and NCI-H520 cells. This finding supported the role of FTO as a potential prognostic factor and therapeutic target [123]. Another study on miRNAs showed the relevance between m6A and apoptosis in ovarian cancer cells. Overexpression of miR-744-5p reduced the enrichment of nuclear factor I X (NFIX) and HNRNPC by modulating their mRNA and protein expression. The former caused decreased Bcl2 levels, exerting proapoptotic effects on cancer cells, whereas the latter downregulated miR-21 and AKT phosphorylation. More importantly, high miR-744 prolonged the median disease-free survival of ovarian serous cystadenocarcinoma patients, indicating the significance of HNRNPC as a prognostic target [126].

Furthermore, overexpression of m6A writer WTAP modulated the AML cell cycle, behaving as G1/S-phase arrest, further inhibiting the cell proliferation and colony formation. Bioinformatics analysis revealed that WTAP knockdown significantly affected PI3K/AKT signaling. On the other hand, WTAP acts as a predictor for poor prognosis in AML. A previous study conducted by Bansal et al. found that deficiency of WTAP upregulated the apoptosis ratio of K562 and HL-60 cells after etoposide challenge [130]. In summary, the prognostic and epigenetic roles of m6A regulators in AML have been extensively investigated. The present studies mainly focus on m6A and AML cell apoptosis, and the interaction between m6A and other PCDs and whether m6A exerts an effect on additional hematopoietic malignances might be an intriguing area of research. Moreover, Wang at el. first demonstrated that the m6A writer RBM15 was overexpressed in laryngeal squamous cell carcinoma (LSCC) and facilitated the progression of LSCC by regulating m6A-based TMBIM6 mRNA, and TMBIM6 m6A modification regulated cell apoptosis. The downregulation of IGF2BP3 inhibited TMBIM6 expression by decreasing its stability, moreover, high RBM15 levels were associated with poor prognosis of LSCC. Taken together, the RBM15/IGF2BP3/TMBIM6 axis could be promising therapeutic and prognostic targets for LSCC patients [139].

Collectively, these results provide us with a research insight: m6A regulators modulate programmed cell death, especially cell apoptosis in diverse cancers. The limited knowledge on the m6A molecule paradigm should inspire researchers to conduct numerous in-depth studies of the role of m6A modifications and PCD in various diseases, which will generate new therapeutic modalities. Therefore, they themselves and their upstream/downstream regulators might act as diagnostic, therapeutic and prognostic biomarkers.

#### Therapeutic resistance

Resistance to therapy strategies (e.g., immunotherapy, radiotherapy and chemotherapy) severely hinder the treatment of cancer patients. Notably, m6A might be considered a potential determinant to cope with therapy resistance through multiple pathways, one of which is the control of downstream adaptive responses (autophagy, apoptosis and the like) [114].

#### Chemotherapy resistance

In gastric cancer cells resistant to chemotherapy, ARHGAP5-AS1 was recognized as an elevated lncRNA, and knocking it down improved chemoresistance. Interestingly, its upregulation was resulted from autophagy inhibition, caused ARHGAP5 transcript activation in the nucleus. Additionally, METTL3 was recruited to motivate the formation of m6A-methylated ARHGAP5 mRNA in the cytoplasm, implying that autophagic degradation of ARHGAP5-AS1 and inhibition of ARHGAP5 could be expected to reverse chemoresistance. As expected, this result produced a promising strategy to improve chemosensitivity and prognosis in gastric cancer by stimulating autophagy of ARHGAP5-AS1 or destabilizing ARHGAP5 caused by METTL3 removal [127]. WTAP, another MTC component, protects natural killer/T-cell lymphoma (NKTCL) cells from Bax-modulated apoptosis induced by cisplatin (DDP) via regulation of dual-specificity phosphatases 6 (DUSP6). That is, ablation of WTAP augmented the chemosensitivity of NKTCL by decreasing the drug resistance-associated proteins MRP1 and P-gp [131]. Saikosaponin-d (SsD), an FTO inhibitor, improved the efficacy of nilotinib and PKC412 by reducing FTO-mediated m^6^A hypomethylation and Bcl-2 transcripts in AML-resistant patients [137]. High m6A, accompanied with TRIM11 overexpression in NPC drug-resistant cells, inhibited apoptosis in vitro and positively regulated the Daple/β-catenin/ABCC9 signaling pathway to impede cisplatin sensitivity. Therefore, reducing m6A levels and further TRIM11 stability will improve chemoresistant NPC therapy [138].

#### Radiotherapy resistance

Radiotherapy is another important treatment method for patients with advanced cancers; however, radioresistance regularly gives rise to treatment failure. Several researches have shown that METTL3 impinges on critical cellular processes including apoptosis to result in resistance to chemo- and radiotherapy in pancreatic cancer cells. Moreover, m6A modifications on lncRNA *MALAT1* could be associated with radioresistance/chemoresistance by impairing the apoptosis response in cervical cancer [128, 129].

#### Immunotherapy resistance

Immune checkpoint blockade (ICB) therapy is a historic revolution in cancer treatment, but only a small percentage of patients respond to it. The mechanism which m6A regulates immunotherapy resistance is under scrutiny. M6A is one of four major RNA adenosine modifications, and their functions are regulated by writers. RNA modification writers were divided into two expression patterns detected by WM_Score: The WM_Score-high group exerted effects on worse patient overall survival and recruitment of inhibitory immune cells and the WM_Score-low group showed effects on a survival advantage, apoptosis, and cell cycle signaling reducing targeted drug resistance. It is worth noting that analysis of the WM_Score could increase the efficacy of PD-L1 blockade by targeting associated writers [140]. The demethylases, FTO and ALKBH5 reduce the response to anti-PD-1 blockade and accelerate melanoma tumorigenesis by reducing m6A methylation of critical protumorigenic melanoma cell-intrinsic genes [103, 142].

Treatment failure arising from primary or acquired resistance is a major hurdle for the survival of cancer patients, particularly advanced patients. Thus, clarifying the molecular mechanisms of therapy resistance in various malignancies is significant for exploring personalized and precise therapeutic approaches. As mentioned above, these findings insinuated that targeting m6A molecules might be an opportunity for resolving therapy resistance.

## Conclusion

M6A is assembled by m6A methyltransferases, recognized by reader proteins, and abolished by m6A demethylases. It remains possible that novel m6A modifiers still need to be identified [143]. Interconnection of apoptosis and ferroptosis, apoptosis and autophagy, and autophagy as well as ferroptosis indicated that m6A could fine-tune more programmed cell death pathways [144, 145]. Programmed cell death by m6A on mRNA and ncRNAs regulates the oncogenesis and development of cancers. However, whether and how m6A proteins and m6A impact PCD remain controversial. For instance, m6A exhibits paradoxical roles—METTL3 serves as an apoptotic driver in HG (high glucose)-reliant human lens epithelial cells (HLECs), while WTAP acts as a suppressor in YTS and SNK-6 cells, two human NKTCL cell lines [146, 147]. There are two main reasons for this discrepancy. One possible explanation is that m6A mRNA status and function are correlated with varied physiological contexts, and the other is the effects of its up/downstream. M6A-associated targets would provide a new direction for clinical diagnosis, treatment, prognosis, and therapy resistance in cancers.

## Data Availability

Not applicable, all information in this review can be found in the reference list.

## Abbreviations

ACS: Acute coronary syndrome
AKI: Acute kidney injury
AMPK: AMP-activated protein kinase
AS: Atherosclerosis
ASC: Apoptotic speck-like protein containing CARD
CAMKK2: Calcium/calmodulin-dependent protein kinase 2
CDS: Coding sequences
DAMPs: Damage-associated molecular patterns
DDP: Cisplatin
DR: Diabetic retinopathy
DUSP6: Dual-specificity phosphatases 6
eIF3: Eukaryotic translation initiation factor 3
ESCs: Embryonic stem cells
FTO: Fat mass and obesity-associated protein
GC: Gastric cancer
GsdmD: Gasdermin-D
HCC: Hepatocellular carcinoma
HG: High glucose
HLECs: Human lens epithelial cells
HNRNPA2/B1: Heterogeneous nuclear ribonucleoprotein A2/B1
HOXA13: Homeobox A13
H/R: Hypoxia/reoxygenation
ICB: Immune checkpoint blockade
IGF2BP1/2/3: Insulin-like growth factor 2 mRNA-binding proteins
IRF: IFN regulatory factor
LCs: Leydig cells
LSCC: Laryngeal squamous cell carcinoma
LSCs: Leukemic stem cells
LUAD: Lung adenocarcinoma
LUSC: Lung squamous cell carcinoma
MALDI-TOF-MS: Matrix-assisted laser desorption/ionization time of flight mass spectrometry
METTL3: Methyltransferase-like protein 3
MTC: Methyltransferase complex
ncRNAs: Noncoding RNAs
NFIX: Nuclear factor I X
NKTCL: Natural killer/T-cell lymphoma
NLRP3: NLR pyrin domain containing 3
NSun2: NOP2/Sun domain family
PA-m6A-seq: Photo-cross-linking-assisted m6A sequencing
PCD: Programmed cell death
PPM1A: Protein phosphatase 1A
PTEN: Phosphatase and tensin homolog
RBM15/15B: RNA binding motif protein 15/15B
RBPs: RNA-binding proteins
R-2HG: R-2-Hydroxyglutarate
R-M: Restriction-modification
SAM: S-adenosyl methionine
SFN: Sulforaphane
SLC3A2: Solute carrier 3A2
SLC7A11: Solute carrier 7A11
SsD: Saikosaponin-d
TAMs: Tumor-associated macrophages
TFs: Transcription factors
TICs: Tumor-initiating cells
TKIs: Tyrosine kinase inhibitors
TME: Tumor microenvironment
UBE2C: Ubiquitin-conjugating enzyme E2C
VIRMA: Vir like m6A methyltransferase associated
WTAP: Wilms tumor 1-associating protein
YAP: Yes-associated protein 1
YTHDC1: YTH domain containing 1
YTHDF1: YTH domain family protein 1
ZC3H13: Zinc finger CCCH-type containing 13
